# Immunoinformatics design of a novel epitope-based vaccine candidate against dengue virus

**DOI:** 10.1038/s41598-021-99227-7

**Published:** 2021-10-05

**Authors:** Adewale Oluwaseun Fadaka, Nicole Remaliah Samantha Sibuyi, Darius Riziki Martin, Mediline Goboza, Ashwil Klein, Abram Madimabe Madiehe, Mervin Meyer

**Affiliations:** 1 Department of Science and Innovation/Mintek Nanotechnology Innovation Centre, Biolabels Node, Department of Biotechnology, Faculty of Natural Sciences, University of the Western Cape, Bellville, South Africa; 2 Plant Omics Laboratory, Department of Biotechnology, Faculty of Natural Sciences, University of the Western Cape, Private Bag X17, Bellville, 7535 Cape Town South Africa; 3 Nanobiotechnology Research Group, Department of Biotechnology, Faculty of Natural Sciences, University of the Western Cape, Bellville, South Africa

**Keywords:** Biochemistry, Biotechnology, Immunology

## Abstract

Dengue poses a global health threat, which will persist without therapeutic intervention. Immunity induced by exposure to one serotype does not confer long-term protection against secondary infection with other serotypes and is potentially capable of enhancing this infection. Although vaccination is believed to induce durable and protective responses against all the dengue virus (DENV) serotypes in order to reduce the burden posed by this virus, the development of a safe and efficacious vaccine remains a challenge. Immunoinformatics and computational vaccinology have been utilized in studies of infectious diseases to provide insight into the host–pathogen interactions thus justifying their use in vaccine development. Since vaccination is the best bet to reduce the burden posed by DENV, this study is aimed at developing a multi-epitope based vaccines for dengue control. Combined approaches of reverse vaccinology and immunoinformatics were utilized to design multi-epitope based vaccine from the sequence of DENV. Specifically, BCPreds and IEDB servers were used to predict the B-cell and T-cell epitopes, respectively. Molecular docking was carried out using Schrödinger, PATCHDOCK and FIREDOCK. Codon optimization and in silico cloning were done using JCAT and SnapGene respectively. Finally, the efficiency and stability of the designed vaccines were assessed by an in silico immune simulation and molecular dynamic simulation, respectively. The predicted epitopes were prioritized using in-house criteria. Four candidate vaccines (DV-1–4) were designed using suitable adjuvant and linkers in addition to the shortlisted epitopes. The binding interactions of these vaccines against the receptors TLR-2, TLR-4, MHC-1 and MHC-2 show that these candidate vaccines perfectly fit into the binding domains of the receptors. In addition, DV-1 has a better binding energies of − 60.07, − 63.40, − 69.89 kcal/mol against MHC-1, TLR-2, and TLR-4, with respect to the other vaccines. All the designed vaccines were highly antigenic, soluble, non-allergenic, non-toxic, flexible, and topologically assessable. The immune simulation analysis showed that DV-1 may elicit specific immune response against dengue virus. Moreover, codon optimization and in silico cloning validated the expressions of all the designed vaccines in *E. coli*. Finally, the molecular dynamic study shows that DV-1 is stable with minimum RMSF against TLR4. Immunoinformatics tools are now applied to screen genomes of interest for possible vaccine target. The designed vaccine candidates may be further experimentally investigated as potential vaccines capable of providing definitive preventive measure against dengue virus infection.

## Introduction

Approximately 12.5% of the world population are affected by a group of pathogens known to cause neglected tropical diseases (NTDs) (CDC, 2020). This diverse group of tropical infections are common among the low-income countries in developing regions of the world. The adverse effects caused by these NTDs include but are not limited to impaired physical and cognitive development, and trapping the poor in a cycle of poverty and disease^1^. Apart from condemning affected people to live with disability and stigma, these NTDs deprive children from attending school, threaten job security, increase the financial burden on countries due to medical cost and in turn negatively affecting the economy of developing countries^2–4^. The ever growing list of NTDs includes, parasitic helminths, bacterial, protozoan, fungal, ectoparasitic and viral diseases. The top 10 hotspots where NTDs dominate include sub-Saharan Africa, Asia, Oceania and the Middle East^5^. For extremely pathogenic NTDs novel therapies are being sought. Although probably less pathogenic or less frequently encountered such as dengue fever, which is neglected despite an apparent increase in prevalence^6^.

The endemic nature of dengue virus has been implicated in more than 100 countries notably in regions of Africa, Americas, Eastern Mediterranean, South-East Asia and the Western Pacific. The largest number of global dengue infection cases ever reported was in 2019 affecting all regions whereas dengue virus transmission was discovered in Afghanistan for the first time. Due to the asymptomatic and mild nature of this disease, the actual number of cases are under-reported with many being misdiagnosed as other febrile illnesses^7^. Dengue is reported as the second most diagnosed cause of fever after malaria and also the dengue fever (DF) being the most rapidly spreading mosquito-borne disease^8^. The rate of infection of dengue virus by Aedes mosquito species (*Aedes aegypti and Aedes albopictus*) increases geometrically with annual infection of about 400 million and 22,000 deaths^9^. Some of the symptoms include high fever accompanied by severe headache, anorexia, abdominal discomfort, maculopapular rash, fatigue, muscle and joint pain, unpleasant metallic taste in the mouth, loss of appetite, vomiting and diarrhea^10,11^. The World Health Organization (WHO) classified DF into uncomplicated and severe DF. In addition, dengue transmission is attributed to four serotypes of the dengue virus (DENV 1–4), where, individuals can be infected by all the four serotypes in their lifetime.

There are several potential dengue vaccines at different stages of development in clinical trial with only one licensed for use. Most of these vaccines are primarily developed from the envelope proteins prM and E, which are expected to elicit protective immune responses in humans^13,14^. Nonetheless, it is essential to consider that the human immune response to DENV is dominated by highly cross-reactive antibodies equipped with neutralizing and enhancing activity^15,16^. The Dengvaxia manufactured by Sanofi Pasteur is a live, attenuated, and tetravalent recombinant vaccine called ChimeriVax^12^. This formulation was the first vaccine to be licensed against dengue in the year 2015 due to its promising results obtained in various clinical studies^17^. In May 2019, Dengvaxia was licensed and approved by the US Food and Drug Administration (FDA) in the United States in areas where dengue was prevalent. This vaccine is available in 19 countries, and its use is limited to for a particular age range and cannot be administered in Flavivírus-naïve individual^18^. Its formulation consists of chimeric viruses constructed by infectious clone technology. Individuals who receive the vaccine and have not been previously exposed to DV may likely develop severe dengue if they are infected after vaccination^19,20^. Although Dengvaxia was evaluated and licensed for human use in 20 countries, it does not contain the non-structural proteins of DENV which presents low protective efficacy and increases their risk of hospitalization^21^. In 2018 the safety issues from the phase III clinical trial of Dengvaxia was published^22^. Due to the increased rate of hospitalization occurring in seronegative vaccinated children between the age of 9–11, Dengvaxia was predicted as vaccine failures rather than as vaccine enhanced dengue disease^23^.

Other vaccine formulations against DENV include live attenuated tetravalent vaccine for dengue (LATV), TAK-003, tetravalent dengue (TDEN), dengue purified inactivated vaccine (DPIV), tetravalent DNA vaccine against dengue (TVDV), and genetic constructs of the tetravalent vaccine formulation (V180)^24^. The LATV was developed by the National Institute of Allergy and Infectious Diseases (NIAID) using recombinant DNA technology^25^. The formulation of this anti-dengue formulation contains the structural and non-structural DENV proteins and the trial study was proven to be safe^26,27^. LATV is currently in phase III clinical trial. The main challenge of LATV is that it requires a significant amount of DENV2 for a balanced seroconversion and the efficacy is yet unknown^28^. Tak-003 produced by Takeda Pharmaceutical Company Limited, is based on an attenuated virus and chimeric viruses constructed using recombinant DNA technology^29^. Currently in phase III clinical trial, Tak-003 in the was shown to be safe, immunogenic, and effective against all the four DENV serotypes with reduced cellular immune response^30,31^. Its efficacy was reported to be 80.2%. However, Tak-003 induces low levels of antibodies to DENV3^32^.

V180 is another anti-dengue potential vaccine registered under the ClinicalTrials.gov is under the phase I clinical trial. It is composed of the recombinant forms of the DENV envelope glycoprotein^33^. Currently, V180 is being developed by Merck and the vaccine formulation is adjuvanted by ISCOMATRIX™^34^. In preclinical study, V180 was proven to be safe and immunogenic due to the high quality of the recombinant proteins and ISCOMATRIX™ adjuvant. Nonetheless, the immunogenic properties of V180 is dependent on adjuvants and three-dose immunization regimen is required^35^. TDEN, DPIV, and TVDV produced by the U.S. Army Medical Research and Materiel Command have been reported to be immunogenic, well-tolerated and safe. While DPIV and TVDV are in phase I clinical trial, TDEN is in phase II clinical trial^36–38^. Challenges associated with these vaccines include general body pain, neutralizing antibody titers decrease over time, TVDV presents only envelop (E) and membrane glycoprotein precursor (M) proteins and requires both adjuvant and high amount of vaccine antigen^39^. Due to the tetravalent nature of DENV, effective vaccine, long-term surveillance for potential adverse events, compatibility with current vaccine schedules, cost and stability are the current challenges in the implementation of the DENV vaccines. Therefore, the improvement in vaccine development against dengue is much needed.

Structurally, the genome of DENV is made of 10,696 nucleotides and 10,173 of these nucleotides are responsible for the single open reading frame (ORF) which encodes a polypeptide of 3,391 amino acid residues^40^. In addition to its single and positive sense RNA stranded (+ ssRNA), it encodes for both structural and non-structural proteins. Immunologically, DENVs are related but genetically and antigenically distinct with approximately 75% relatedness^41,42^. DENV serotypes are subdivided according to the envelope gene responsible for incomplete cross-defensive immunity towards other serotypes in *Homo sapiens*^43^. As perceived by the immune system, disease development, viral replication and antigenic determinants have been linked with DENV proteins^44,45^. Therefore, any antigenic DENV protein sequences that is fit to elicit immune responses its host could be employed in the identification of vaccine for dengue^46^.

Asides palliative therapy, the treatment of both dengue and severe dengue are not specific and the mode of action by which the immune system react to this infection is unclear. Moreover, successful mosquito control measure to limit the spread of DF is still in its infant stage. As such, epitope-based vaccine development remains a clear solution for this infection.

In this study, several computational approaches were applied to design multiple epitope-based vaccines against DENV that comprises multiple epitope, which elicit the activation of cytotoxic T lymphocytes (CTLs) and helper T lymphocytes (HTLs). These vaccines require simple peptides that are easily chemically synthesized rather than pathogens. These approaches present an advantage over conventional methods of vaccine development by reducing the chance of allergenic reactions. In addition, these methods are rapid, easy and cost-effective. Although, similar approaches have been widely employed for the construction of vaccines against viruses such as SARS-CoV-2^47^, Ebola^48^, Zika^49^, and Chikungunya^50^, to date, no commercial and licensed epitope-based vaccine are available on the market using these bioinformatics approaches^51,52^.

## Results

### Sequence selection

In order to develop an epitope/ multi-epitope based vaccine for DEN-4, the protein sequences of DENV (structural and non-structural sequences) were obtained from NCBI and UniProt. The process of component sequence selection was modified from Ali et al.^53^. These components are presented in Table 1. Their physical and chemical properties are also presented in Table 2. Structural proteins are responsible for host invasion and particle assembly. On the other hand, enzyme synthesis which aids viral replication and structural protein synthesis are attributed to non-structural proteins. After infection, they prompt different immune responses. Prior to downstream analysis, the retrieved sequences were subjected to antigenicity and transmembrane helices prediction, and physicochemical analysis. All the three structural proteins were confirmed antigenic at a threshold of 0.4. While the non-structural components NS2A, NS2B and NS4A were non-antigenic due to their low prediction score, NS1, NS3 and NS5 were predicted to be antigenic. With a prediction score of 0.544, non- structural NS5 was the most antigenic viral component of DEN-4 (Table 3).

**Table 1:** Structural and non-structural protein sequences of DENV 4 retrieved from NCBI.

RefSeq	Protein name	Length (aa)	Accession ID
NP_073286.1	Polyprotein	3387	NP_073286
NP_740313.1	Capsid protein (C)	99	NP_740313
NP_740315.1	Membrane glycoprotein precursor (M)	166	NP_740315
NP_740317.1	Envelope protein (E)	495	NP_740317
NP_740318.1	Nonstructural protein NS1	352	NP_740318
NP_740319.1	Nonstructural protein NS2A	218	NP_740319
NP_740320.1	Nonstructural protein NS2B	130	NP_740320
NP_740321.1	Nonstructural protein NS3	618	NP_740321
NP_740322.1	nonstructural protein NS4A	127	NP_740322
NP_740324.1	Nonstructural protein NS4B	245	NP_740324
NP_740325.1	RNA-dependent RNA polymerase NS5	900	NP_740325

**Table 2:** Physicochemical properties of the viral protein.

Parameters	prM	E	NS1	NS2A	NS2B	NS3	NS4A	NS4B	NS5
Total AAs	166	495	352	218	130	618	127	245	900
Mol. w	18,709.67	53,988.10	39,635.01	24,039.33	13,939.24	69,397.18	13,910.58	26,580.16	102,969.54
Theo. pI	6.35	7.90	6.55	9.72	4.27	8.35	5.26	9.01	8.80
Ext. coeff	33,835	72,140	96,660	19,730	26,470	99,600	13,980	36,565	214,125
Half-life	1.1	30	1.1	30	1.9	1.9	1.9	1.4	30
I.I	47.04	24.64	44.12	48.82	29.02	33.53	34.42	28.62	38.21
Alip. index	75.78	80.67	67.84	131.88	116.23	78.11	132.20	105.59	71.13
GRAVY	− 0.122	− 0.72	− 0.460	0.852	0.422	− 0.479	0.667	0.273	− 0.607

https://web.expasy.org/cgi-bin/protparam/protparam.

**Table 3:** Antigenicity prediction of dengue virus proteins. (Threshold = 0.4 with tumor model).

Viral component	VaxiJen score	Probability	Localization
Capsid protein	0.4938	Antigenic	Mitochondria
	0.4725	Antigenic	Plasma membrane
Envelope protein	0.4090	Antigenic	Cytoplasmic
Nonstructural protein NS1	0.4340	Antigenic	Extracellular
Nonstructural protein NS2A	0.0365	Non-antigenic	Plasma membrane
Nonstructural protein NS2B	0.1671	Non-antigenic	Plasma membrane
Nonstructural protein NS3	0.4756	Antigenic	Mito, Cytoplasmic
Nonstructural protein NS4A	0.0957	Non-antigenic	Plasma membrane
Nonstructural protein NS4B	0.1549	Non-antigenic	Plasma membrane
Nonstructural protein NS5	0.54368	Antigenic	Cytoplasmic

The TMHMM Server DTU Bioinformatics prediction tool revealed the position and amino acid range of the sequences of the selected viral components (Table 4). The capsid protein and selected sequence length of the envelop protein (466–471) were predicted to be localized inside the cell. All selected antigenic non-structural protein components and membrane glycoprotein precursor sequences were localized outside the cell.

**Table 4:** Prediction of transmembrane helices in proteins using the TMHMM Server v2.0.

Viral component	Position	Amino acids
Capsid protein	Inside	1–99
Membrane glycoprotein precursor	Outside	1–166
Envelope protein	Outside	1–442
	TMhelix	443–465
	Inside	466–471
	TMhelix	472–494
	Outside	495–495
Nonstructural protein NS1	Outside	1–352
Nonstructural protein NS3	Outside	1–618
Nonstructural protein NS5	Outside	1–900

### Prediction of B-cell epitope

B-cell epitopes were predicted using BCPREDS for all antigenic structural and non-structural protein components of DENV-4. Predicted epitopes were shortlisted on the basis of prediction score, toxicity, allergenicity, antigenicity, hydrophobicity and surface accessibility. All epitopes with a score ≥ 0.70 were selected for vaccine design. A total of 47 epitopes were predicted for all protein components while only 9 epitopes satisfied the set criteria and were selected for further analysis. One epitope each was predicted for the capsid protein, membrane glycoprotein precursor and NS1. Although no B-cell epitope was predicted for the confirmed antigenic sequences of NS3, 4 and 2 B-cell epitopes were shortlisted for NS5 and the envelop components respectively. These selected epitopes were further validated using BepiPred for their structural and relative surface accessibility through NetsurfP's default threshold. Regions of the B-cell epitopes (italicized) were predicted by SVMTrip with a score of 1.00 (Table 5).

**Table 5:**
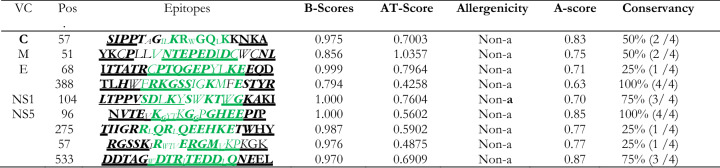
Selected B-cell epitopes.

The B-cell epitopes were predicted using BCPREDS web-based tool and were validated by BepiPred 2.0 software. Epitopes are represented as BCPREDS predicted. Green colored epitopes were predicted by BepiPred. Additionally, underlined amino acids are structurally coiled whereas the amino acids (not underlined) are structurally helix. For surface accessibility, subscripted amino acids are buried as against the exposed amino acids. *VC* viral component; *Pos.* position; *B-score* BCPred score, *A-score* ABCpred score, *AT-score* antigenicity score. For antigenicity prediction, the threshold was set to 0.4, which implies that any predicted epitope with antigenicity score ≥ 0.4 is said to be antigenic. In addition, regions of the B-cell epitopes predicted by SVMTrip with a score of 1.00 were italicized.

### T-cell epitopes predictions and prioritization

Table 6 shows the final list of the selected epitopes for each of the antigenic viral components. Briefly, the selected viral components of DV were subjected to various MHC I and II epitopes prediction web-based servers (immune epitope database and analysis resources as well as the CTLPred). T cell epitopes (HTL and CTL) were ranked based on stringent in-house criteria before being shortlisted for downstream analysis. These rules of selection include: Good IEDB score, high conservancy, good binding affinity, B-cell epitope overlap, ≥ 9mer for MHC I, 15mer for MHC II, significantly antigenic/immunogenic and topographically accessible to membrane-bound or free antibody. For high immunogenicity, lower percentile ranks and IC_50_ value were considered. According to these specifications, 21 epitopes each (C-2, M-3, E-4, NS1-1, NS3-3, and NS5-8) and (C-5, E-5, NS1-1, NS3-5, and NS5-5) were shortlisted for MHC-I and MHC II binders for multi-epitope based vaccine designed.

**Table 6:** Composite table of the prioritized epitopes for vaccine development.

	MHC I	MHC II	B-Cell
C	VVRPPFNMLK SIPPTAGILK	FLRVLSIPPTAGILK KEIGRMLNILNGRKR LRVLSIPPTAGILKR PFNMLKRERNRVSTP FNMLKRERNRVSTPQ	***SIPPT***_*A*_***G***_*IL*_***K*R**_W_**GQ**_L_**KKNKA**
M	LLFKTTEGINK GTCTQSGER VAKHERGRPLLFK		**YK***C****P****LLV****NTEPED****I****D****CWC****NL***
E	VTFKVPHAK MSYTMCSGK VTFKVPHAKR TTAKEVALLR	NHGVTAMITPRSPSV HGVTAMITPRSPSVE HKQWFLDLPLPWTAG YKERMVTFKVPHAKR KQWFLDLPLPWTAGA	**I*TTATR****C****PTQGEP****YL****KEEQ*D** **TL*H****WF****RKGSS****IG****K****MF****ESTYR***
NS1	LMSAAIKDQK	EQYKFQPESPARLAS	***LTPPVSD****L****K****Y****S****W****KT****W****GKA*KI**
NS3	KVASAGISY HVQTKPGLFK VQTKPGLFK	AAIFMTATPPGATDP AIFMTATPPGATDPF KSGDYVSAITQAERI SGDYVSAITQAERIG TKSGDYVSAITQAER	
NS5	ATLKNVTEVK YVDYMPVMK RGAVMDIISRK LTYQNKVVK RSVSTETEK KVDTRTPQPK CTREEFISK WSYYMATLK	LGKAYAQMWSLMYFH TREEFISKVRSNAAI CLGKAYAQMWSLMYF PWDVIPMVTQLAMTD ACLGKAYAQMWSLMY	**N*VTE***_*V*_***K***_***G****YT*_*K****G***_***G***_*P****GHEEPI*P** ***TIIGRR***_*L*_***QR***_*L*_***QEEHKETW*HY** ***RGSSK***_*I*_***R***_*WIV*_***ERGM***_*V*_*KPK*GK ***DDTAG***_*W*_***DTR***_*I*_***TEDD***_*L*_***QNE*EL**

Underlined residues are structurally coiled whereas the amino acids (not underlined) are structurally helix. For surface accessibility, subscripted amino acids are buried as against the exposed amino acids. In addition, regions of the B-cell epitopes predicted by SVMTrip with a score of 1.00 were italicized.

### Immune receptors

**Figure 1:**
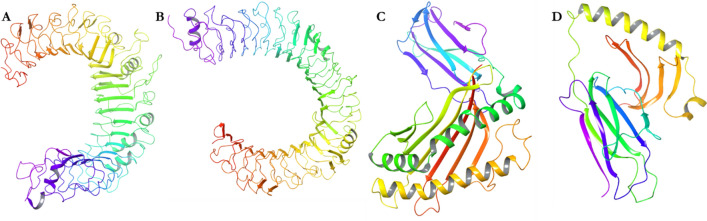
The prepared 3D structures of the Toll-like and Major histocompatibility complex receptors used in this study. (**A**) TLR-2; PDB ID: 2ZX; (**B**) TLR-4; PDB ID: 3FX1; (**C**) MHC class I; PDB ID: 5WJL; and (**D**) MHC class II; PDB ID: 5JLZ.

The receptors considered for docking include two immune alleles (HLA-A*11-01 and HLA-DRB1*04-01) and two immune molecules toll-like receptors (TLR2 and TLR4). Their respective crystallographic structures with PDB IDs (HLA-A*11-01: 5WJL and HLA-DRB1*04-01: 5JLZ; TLR2: 3FXI and TLR4: 2Z7X) were retrieved from the protein data bank. Since proteins from the PDB database often lack coordinates for side-chains on the surface of the protein given the complexity to determine side chain coordinates from x-ray crystallographic data, all proteins were refined through the protein preparation wizard in Schrödinger to improve the overall quality of the output. These refined outputs (Fig. 1) were then used for docking analysis.

### Vaccine construction

From the prioritized epitopes, four vaccines were constructed, with the aim to elicit an immune response against DENV-4. Three different adjuvants i.e. beta defensin, L7/L12 ribosomal protein and HABA protein were used to construct four different vaccines. PADRE sequence is a pan HLA-DR epitope peptide that was used for vaccine construction to enhance their potency with minimal toxicity. The PADRE sequence improves the CTL response as it has the capability to bind with different MHC class-II molecules with high binding affinity (Wu et al., 2010). Also, appropriate linkers were used for the conjugation of the prioritized epitopes during the design process. The final vaccine lengths for DV1-4 were 352, 336, 336 and 400 respectively (Table 7).

**Table 7:** Predicted multi-epitope based vaccines.

Vaccine name	Length	Vaccine constructs
Dengue virus vaccine-1 (DV-1)	352	GIINTLQKYYCRVRCAVLSCLPKEEQIGKCSTRGRKCCRRKKEAAAK**AKFVAAWTLKAAA**GGGSVVRPPFNMLKAAYLLFKTTEGINKAAYVTFKVPHAKAAYLMSAAIKDQKAAYKVASAGISYAAYATLKNVTEVKGGGSFLRVLSIPPTAGILKGPGPGNHGVTAMITPRSPSVGPGPGEQYKFQPESPARLASGPGPGAAIFMTATPPGATDPGPGPGLGKAYAQMWSLMYFHGGGS*SIPPTAGILK*RWGQLKKNKAKKYK*CPLLVNTEPEDIDCWCNL*KKI*TTATRCPTQGEPYLKEEQ*DKK*LTPPVSDLKYSWKTWGKA*KIKKN*VTEVKGYTKGGPGHEEPI*P
Dengue virus vaccine-2 (DV-2)	336	MAKLSTDELLKEMTLLELSDFVKKFEETFEVTAAAPVAVAAAGAAPAGAAVEAAEEQSEFDVILEAAGDKKIGVIKVVREIVSGLGLKEAKDLVDGAPKPLLEKVAKEAADEAKAKLEAAGATVTVKEAAAK**AKFVAAWTLKAAA**GGGSSIPPTAGILKAAYGTCTQSGERAAYMSYTMCSGKAAYHVQTKPGLFKAAYYVDYMPVMKGGGSKEIGRMLNILNGRKRGPGPGHGVTAMITPRSPSVEGPGPGAIFMTATPPGATDPFGPGPGTREEFISKVRSNAAIGGGSTL*HWFRKGSSIGKMFESTYR*KK*TIIGRRLQRLQEEHKETW*HY
Dengue virus vaccine-3 (DV-3)	336	MAENPNIDDLPLAALGAADLALATVNDLIANLRERAEETRAETRTRVEERRARLTKFQEDLPEQFIELRDKFTTEELRKAAEGYLEAATNRYNELVERGEAALQRLRSQTAFEDASARAEGYVDQAVELTQEALGTVASQTRAVGERAAKLVGIELEAAAK**AKFVAAWTLKAAA**GGGSVAKHERGRPLLFKAAYVTFKVPHAKRAAYVQTKPGLFKAAYRGAVMDIISRKGGGSLRVLSIPPTAGILKRGPGPGHKQWFLDLPLPWTAGGPGPGKSGDYVSAITQAERIGPGPGCLGKAYAQMWSLMYFGGGS*RGSSKIRWIVERGMVKPK*GK
Dengue virus vaccine-4 (DV-4)	400	MSDINKLAENLKIVEVNDLAKILKEKYGLDPSANLAIPSLPKAEILDKSKEKTSFDLILKGAGSAKLTVVKRIKDLIGLGLKESKDLVDNVPKHLKKGLSKEEAESLKKQLEEVGAEVELKEAAAK**AKFVAAWTLKAAA**GGGSTTAKEVALLRAAYLTYQNKVVKAAYRSVSTETEKAAYKVDTRTPQPKAAYCTREEFISKAAYWSYYMATLKGGGSPFNMLKRERNRVSTPGPGPGFNMLKRERNRVSTPQGPGPGYKERMVTFKVPHAKRGPGPGKQWFLDLPLPWTAGAGPGPGSGDYVSAITQAERIGGPGPGTKSGDYVSAITQAERGPGPGPWDVIPMVTQLAMTDGPGPGACLGKAYAQMWSLMYGGGS*DDTAGWDTRITEDDLQNE*EL

The distinct bold sequences are the pan HLA DR-binding epitope (PADRE) while the B cell epitopes are italicized.

### Designed vaccine properties analysis

The various vaccine components including their specific properties are presented in Table 3. In addition to the different adjuvants used for the design, the antigenicity, allergenicity, toxicity and surface accessibility of these vaccines were also evaluated (Table 8). All designed vaccines are antigenic in nature, non-allergenic and non-toxic. Furthermore, their surface accessibilities were predicted to be localized outside of the cell.

**Table 8:** Composition and assessment of the primary sequence of the multi-epitope based vaccine construct.

Vaccines	Adjuvant used	Number of HTL	Number of CTL	Number of B cell	Length	Antigenicity	Allergenicity	Toxicity	Surface accessibility
DV-1	BD	6	5	5	352	0.6136	NA	NT	Outside
DV-2	RP	5	4	2	336	0.6184	NA	NT	Outside
DV-3	HBHA	4	4	1	336	0.6945	NA	NT	Outside
DV-4	RP	6	8	1	400	0.6764	NA	NT	Outside

*BD* beta defensing, *RP* ribosomal protein, *HBHA* Heparin-binding hemagglutinin; *NA* non-allergen, *NT* non-toxic.

The physicochemical properties of these vaccines were computed using various web-based servers. Parameters evaluated include; solubility, amino acid composition (AAS), molecular weight Mol. W), theoretical pI (Theo. pI), extinction coefficient (Ext. coeff), half-life, instability index (I.I), aliphatic index (A.I), and the grand average of hydropathicity (GRAVY) (Table 9). DV-1 had the highest predicted solubility and Theo. pI of 0.636 and 9.87 respectively. However, DV-4 had the highest AAs sequence and predicted ext. coeff of 400 and 59,360 M^−1^ cm^−1^ respectively. All of the designed vaccines were found to have similar predicted half-life of 30 h. However, DV-3 had the highest predicted aliphatic index (82.08) while DV-2 had the highest GRAVY of -0.167 among the designed vaccines. However, further laboratory studies are required to investigate the accuracy of these results.

**Table 9:** Physicochemical properties of the predicted multi-epitope based vaccine construct.

Parameters	DV-1	DV-2	DV-3	DV-4
Solubility	0.636	0.555	0.556	0.597
No. of amino acids	352	336	336	400
Molecular weight	37,586.86	35,295.60	36,255.51	42,958.22
Theoretical pI	9.87	9.14	9.68	9.72
Ext. coeff	55,975	29,910	40,910	59,360
Half-life	30 h	30 h	30 h	30 h
Instability Index	36.22	38.17	33.45	29.34
Aliphatic index	68.58	75.39	82.08	76.17
GRAVY	− 0.376	− 0.167	− 0.331	− 0.449

### Secondary structural prediction and validation of the designed vaccines

**Figure 2:**
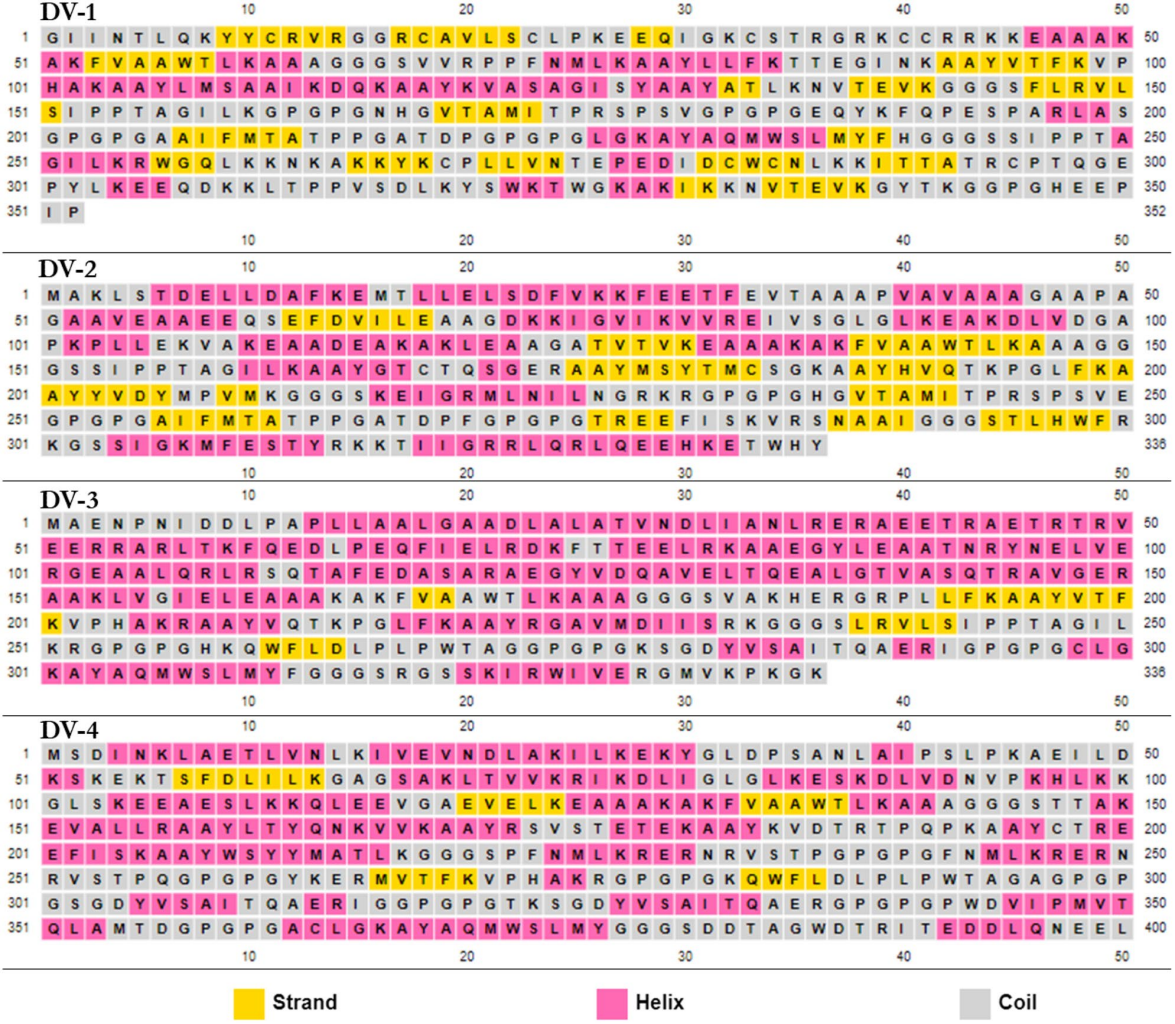
The secondary structures of the predicted dengue virus vaccines (PSIPRED).

**Figure 3:**
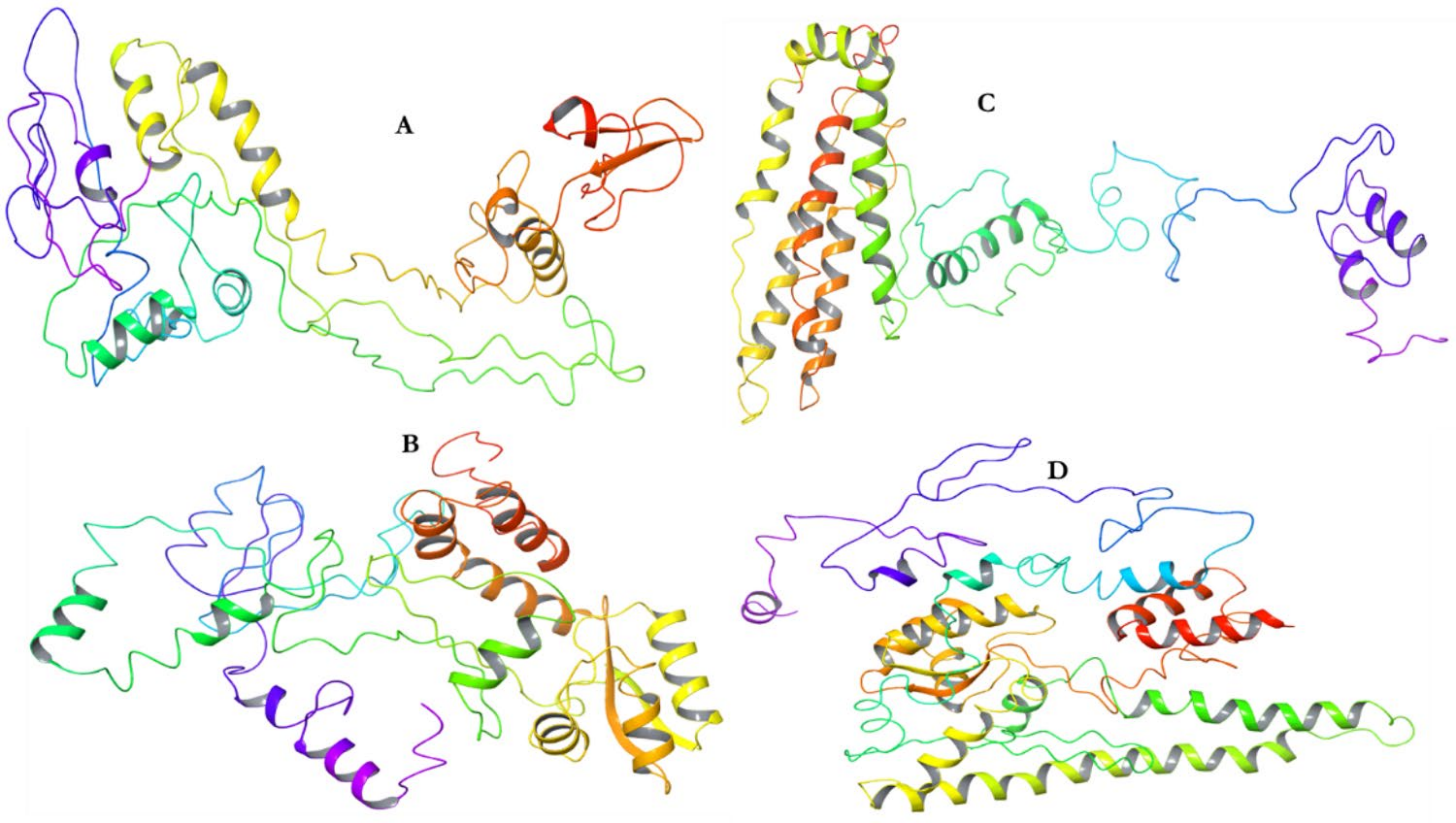
The predicted multi-epitope vaccines for DENV. A) DV-1; B) DV-2; C) DV-3; and (**D**) DV-4 as modelled by Schrödinger.

The secondary structure of the four designed vaccines were predicted by PSIPRED (Fig. 2). The structures are composed of coils, helixes and strands with no structural deformities. Epitopes often are found in coils and needs to be exposed. The sequences were then modelled by several prediction tools, including Schrödinger, to ensure accuracy (Fig. 3). The knowledge-based method of homology modeling of prime module was employed under Schrödinger software. For DV-1, the template ID: 2LX0 with percentage identity of 58% and expected value (e-value) of 1 × 10^–8^ was taken into consideration. For DV-2 to 4, the templates PDB IDs include 1DD3, 3J00, and 3J7Z with % identity of 84%, 58% and 43%, respectively. The e-values of DV-2 to 4 were 2.4 × 10^–16^, 1.2 × 10^–2^, and 6.0 × 10^–21^, respectively. The coverage of these modeled templates are 37%, 39%, 35% and 30%, respectively for DV-1 to 4.

**Figure 4:**
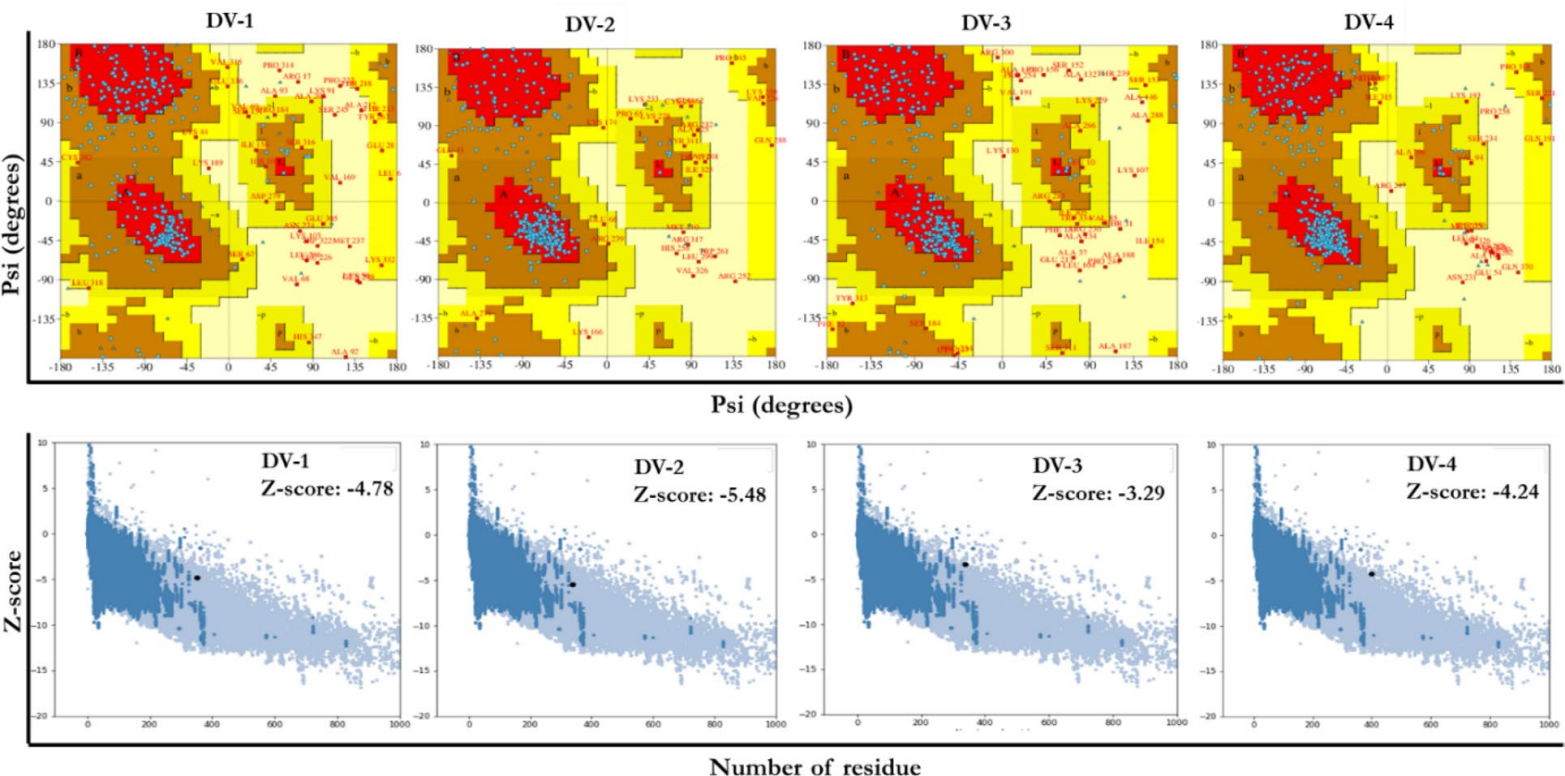
Validation of the modeled-predicted DENV vaccines with ProSA-Web.

The modelled outputs were further validated by PROCHECK and ProSA-web servers prior to docking analysis (Fig. 4).

### Docking study of the designed vaccines against the immune molecules

**Figure 5:**
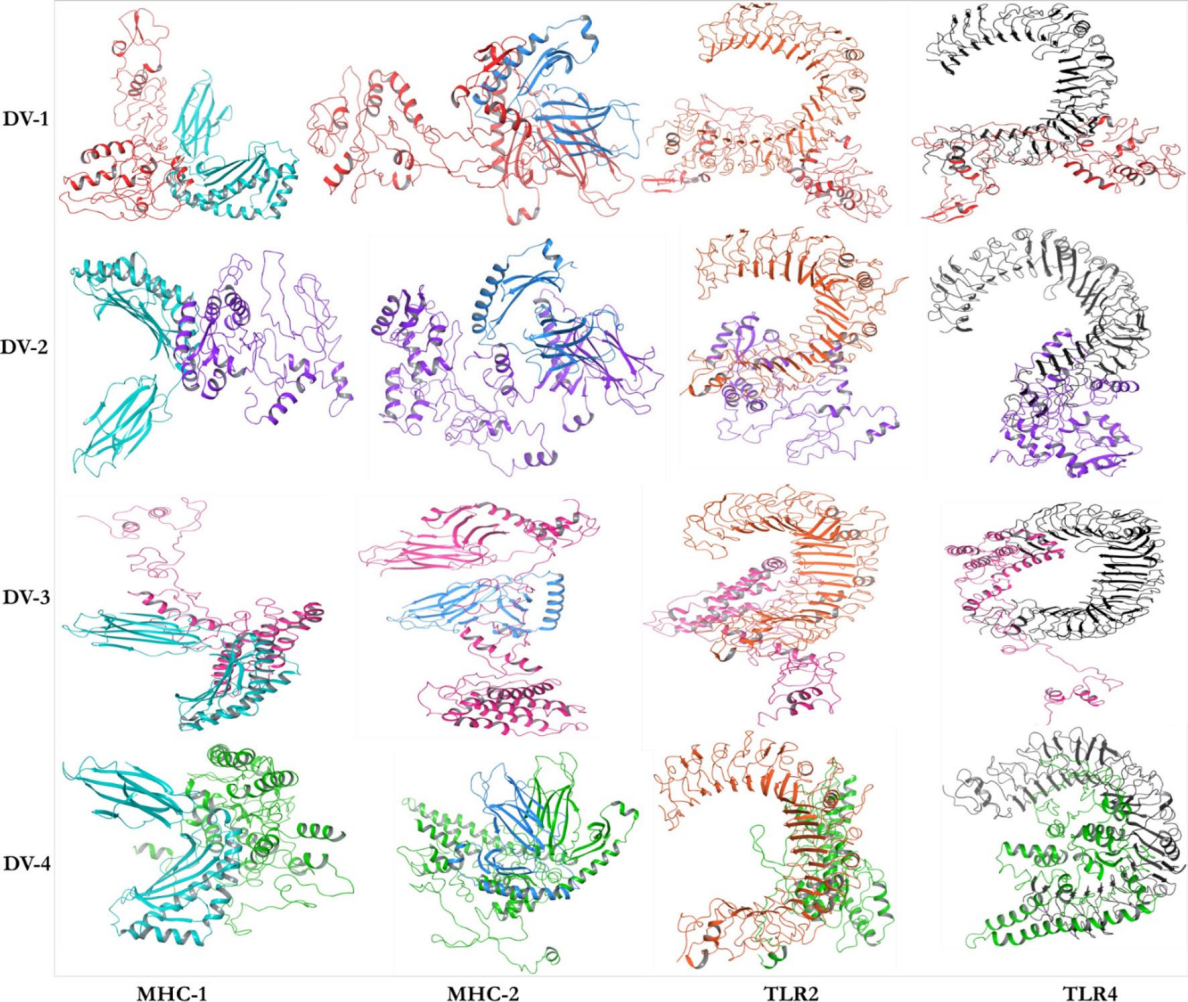
Docking poses of the predicted DENV vaccines against selected immunological receptors (Schrodinger suite v2020-3).

**Figure 6:**
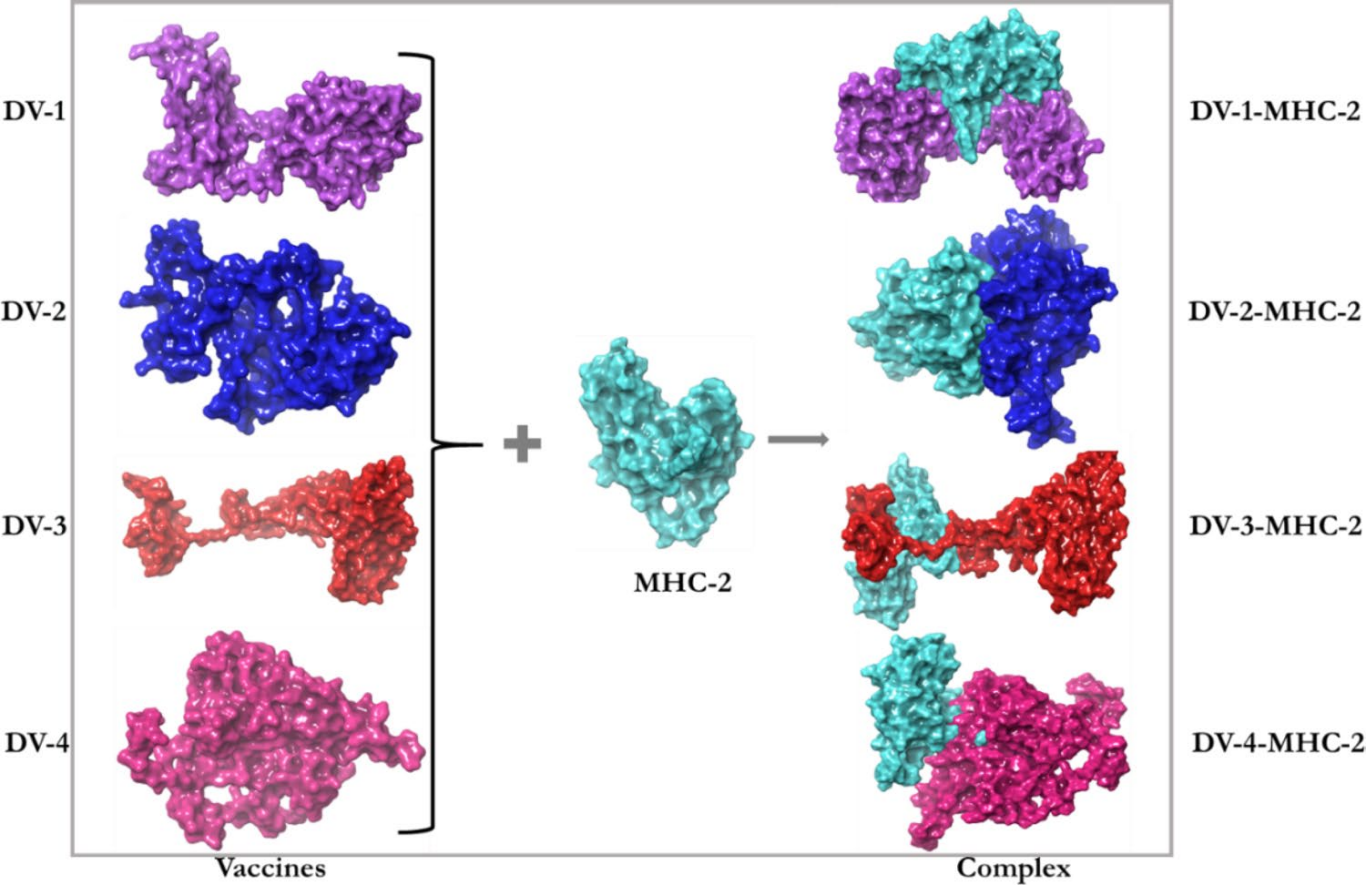
The solid molecular surface display of the predicted vaccines docked to the MHC class-2 receptor.

**Figure 7:**
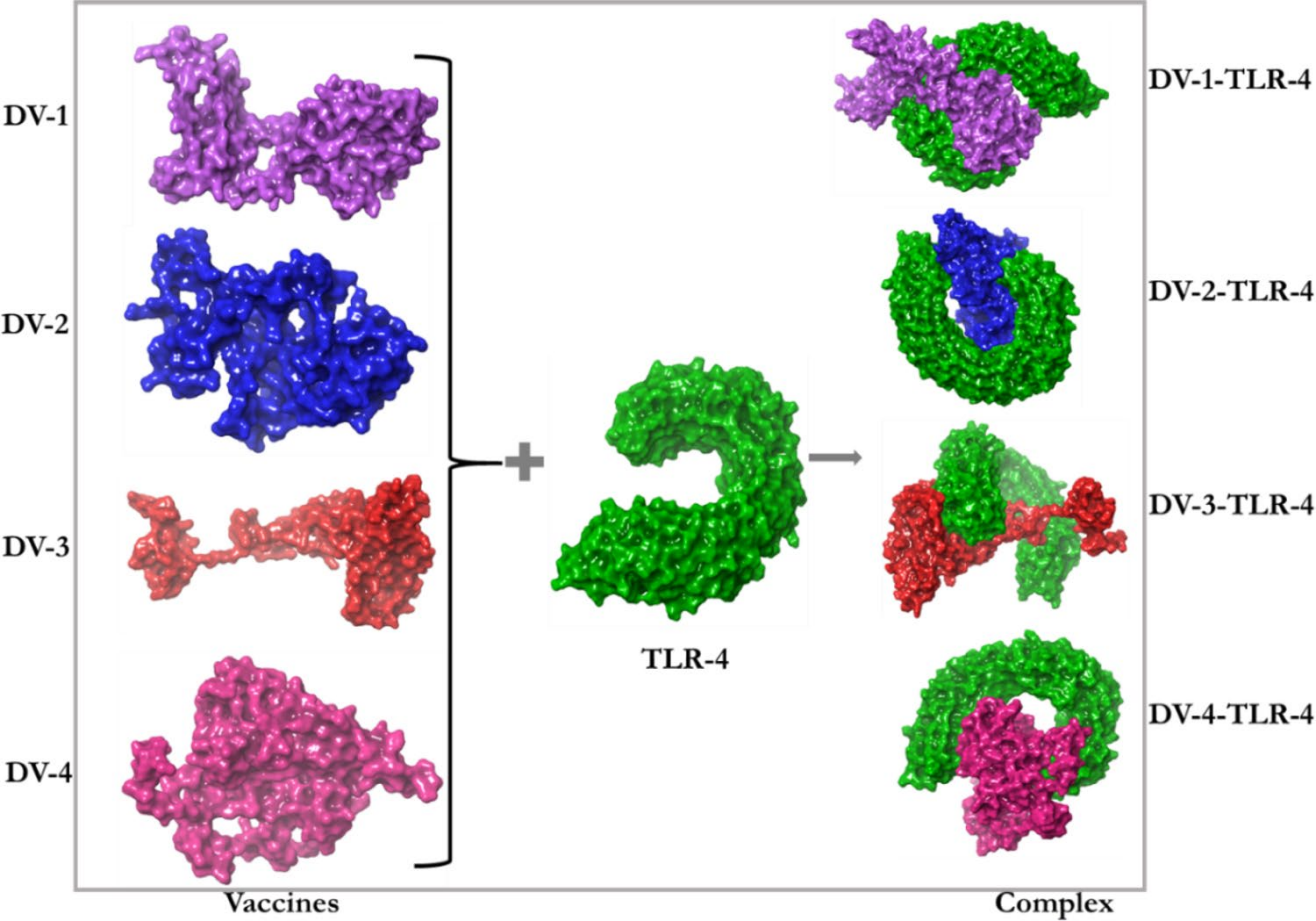
The solid molecular surface display of the predicted vaccines docked to the TLR-4 receptor.

The interactions between an antigenic molecules and an immune receptors molecules are crucial to effectively transport the antigenic molecule and to activate immune response^54,55^. Therefore, docking analysis was carried out between the immune receptor molecules (MCHs and TLRs) and the designed vaccines (DV-1–4) in order to investigate possible interactions, binding energy and poses. Docking analysis was carried out using Schrödinger as well as PATCHDOCK and FIREDOCK to enhance the accuracy of the prediction (Fig. 5). Schrödinger was specifically used to evaluate the generated poses including their solid molecular surface display (Figs. 6 and 7)^56^. The PATCHDOCK server was used to generate the binding scores, area and ACE. The top 100 poses were refined using the FIREDOCK server where the global and hydrogen bond energies were generated for the docking calculation (Table 10). TLRs can efficiently induce the immune response after virus recognition. For TLRs, DV-1 showed the best binding among the four designed vaccines for TLR2 and TLR4 with binding energies of − 63.40 kcal/mol and − 69.89 kcal/mol. Respectively, followed by DV-4 (− 52.53 kcal/mol) and DV-3 (− 60.23 kcal/mol). For the MHC alleles, DV-1 and DV-4 showed the best binding interactions with HLA-A*11–01 and HLA-DRB1*04–01 with global energies of − 60.07 kcal/mol and − 67.22 kcal/mol. respectively. For these alleles, DV-4 and DV-3 also showed lower energies compared to DV-2. The docking analysis showed strong interactions as well as effective binding between the designed vaccines and MCH1, MCH2, TLR2 and TLR4. The docking statistics are shown in Table 10.

**Table 10:** The binding properties of the docking analysis using PATHDOCK and refined by FIREDOCK.

Vaccine construct	Receptor	Name of target	Score	Area	H-bond E	Global E	ACE
DV-1	5WJL	HLA-A*11–01	10,204	14.12.80	− 4.81	− 60.07	− 114.00
	5JLZ	HLA-DRB1*04–01	10,056	1240.30	− 2.19	− 51.02	− 140.36
	3FXI	TLR2	15,766	2591.40	− 3.20	− 63.40	− 33.79
	2Z7X	TLR4	16,202	1880.60	− 5.73	**− 69.89**	103.14
DV-2	5WJL	HLA-A*11–01	9098	1523.60	− 1.90	− 48.11	145.04
	5JLZ	HLA DRB1*04–01	9072	1387.70	− 1.24	− 59.30	− 89.11
	3FXI	TLR2	15,784	2264.40	− 1.14	− 47.31	162.29
	2Z7X	TLR4	17,282	2301.00	− 1.69	− 50.15	13.11
DV-3	5WJL	HLA-A*11–01	10,162	1642..20	− 2.51	− 41.64	307.70
	5JLZ	HLA DRB1*04–01	10,740	1373.90	− 3.83	− 63.84	− 111.34
	3FXI	TLR2	15,328	1816.70	− 4.09	− 45.59	182.27
	2Z7X	TLR4	15,214	1893.10	− 2.17	− 60.23	121.10
DV-4	5WJL	HLA-A*11–01	10,444	2290.20	− 3.85	− 57.16	256.86
	5JLZ	HLA DRB1*04–01	10,350	1326.40	− 3.48	**− 67.22**	− 196.17
	3FXI	TLR2	15,726	2291.70	− 5.90	− 52.53	− 99.35
	2Z7X	TLR4	15,420	3052.30	− 6.31	− 50.39	464.55

*H-Bond E* Hydrogen bond energy; *Global E* Global energy, *ACE* Atomic contact energy

*Allelic variants.

### Optimization and in silico cloning of the designed vaccines

**Figure 8:**
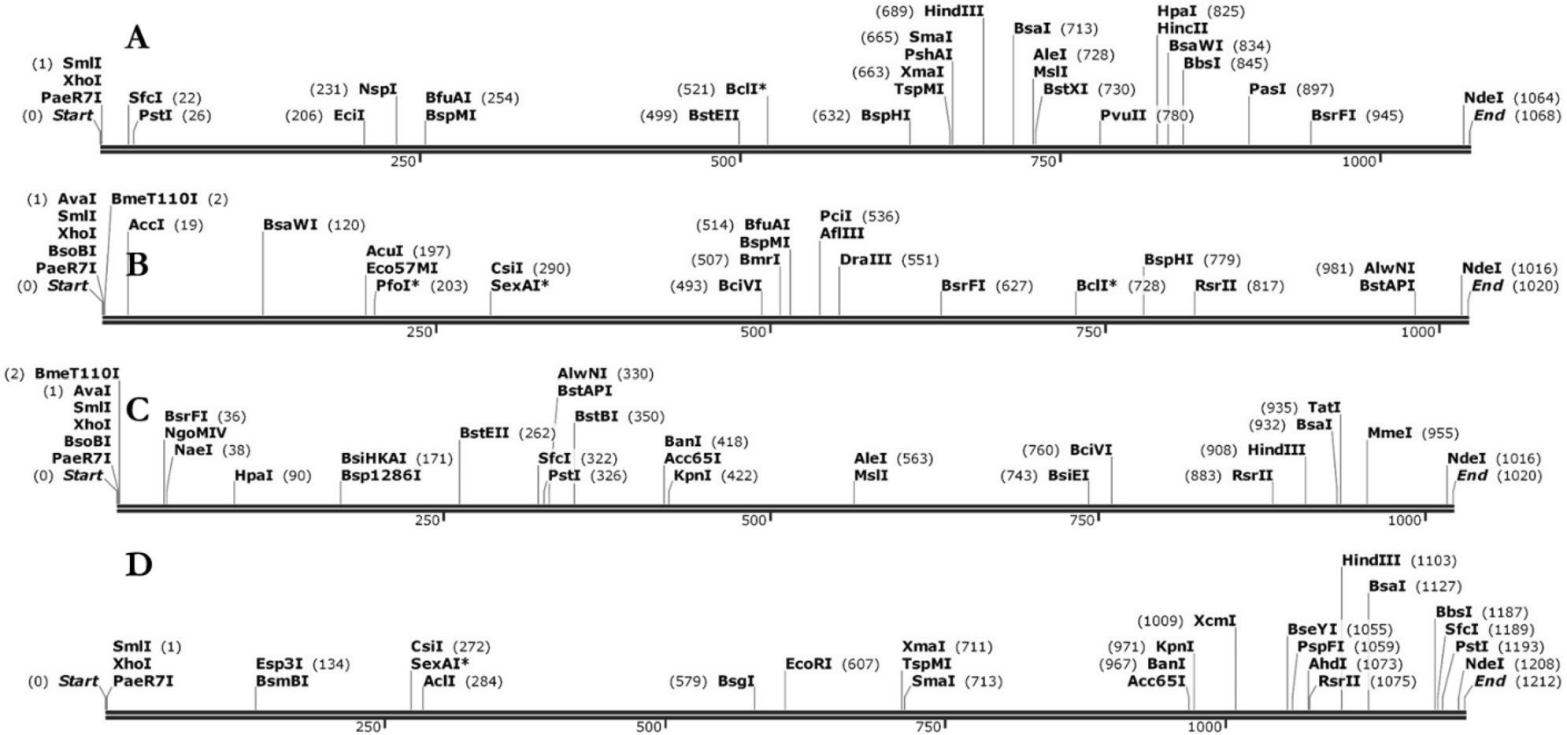
The linear vaccine constructs with *Xho*I and *Nde*I sites. (**A**) DV-1, (**B**) DV-2, (**C**) DV-3, and (**D**) DV-4 (SnapGene software).

**Figure 9:**
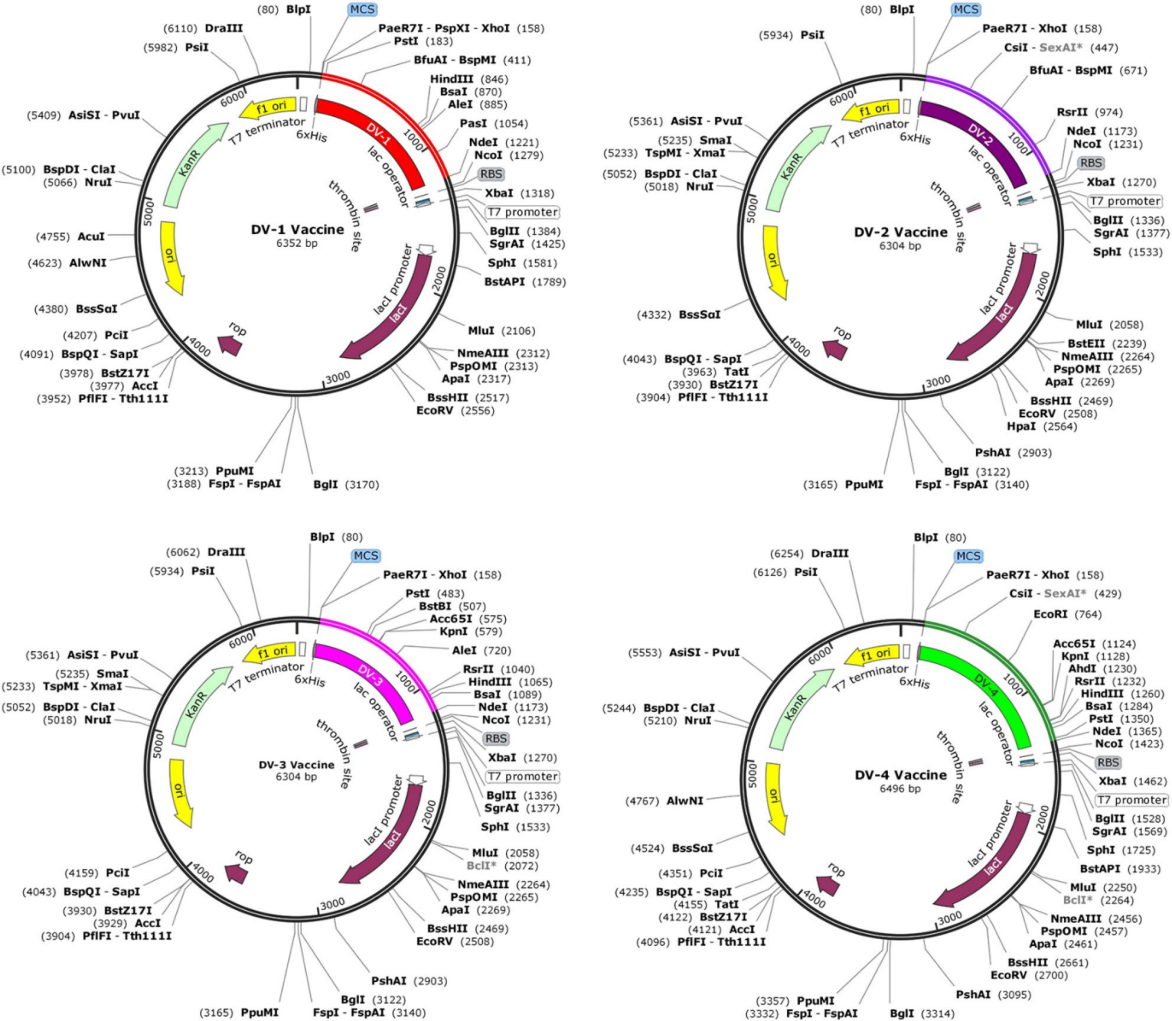
In silico restriction cloning of the codon optimized final designed vaccines in the pET-28a (+) vector between the *Xho*I (158) and *Nde*I (238) restriction enzyme sites (SnapGene software). The final constructs can further be expressed in *E coli* (strain K12) for efficient vaccine production.

Efficient expression of vaccines into *E. coli* expression system is a crucial step in in silico cloning. Four vaccines (DV-1–4) were designed with the prioritized B-cells and T-cell predicted epitopes with appropriate adjuvants and linkers. The sequences of these vaccines were used as input (individually) in the Java codon adaptation tool (JCAT) in order to adapt the codon usage to most sequenced prokaryotic organisms. The result of this analysis shows that the DNA sequences of DV-1–4 were 1056, 1008, 1008 and 1200 nucleotides respectively. The observed CAIs (DV-1 = 1.0; DV-2 = 1.0; DV-3 = 0.99 and DV-4 = 0.98) indicated that the adapted sequences were made up of codons capable of cellular machinery of the target organism. Furthermore, the GC content of the improved sequences were in the range of 50.99% to 54.27%. This sequence information implies the efficient expression and reliability of the designed vaccines in *E. coli*. Restriction recognition sites of the restriction enzymes *Xho*I and *Nde*I were conjugated at the N and C-terminal of reverse translated nucleotides of DV-1–4 respectively (Fig. 8) and the final optimized sequences were inserted into the pET28a (+) vector in order to clone the designed vaccines using SnapGene software. The final lengths of the cloned plasmids were 6.352, 6.304, 6.304 and 6.396 kbp for DV-1–4, respectively (Fig. 9).

### Immune simulation analysis

**Figure 10:**
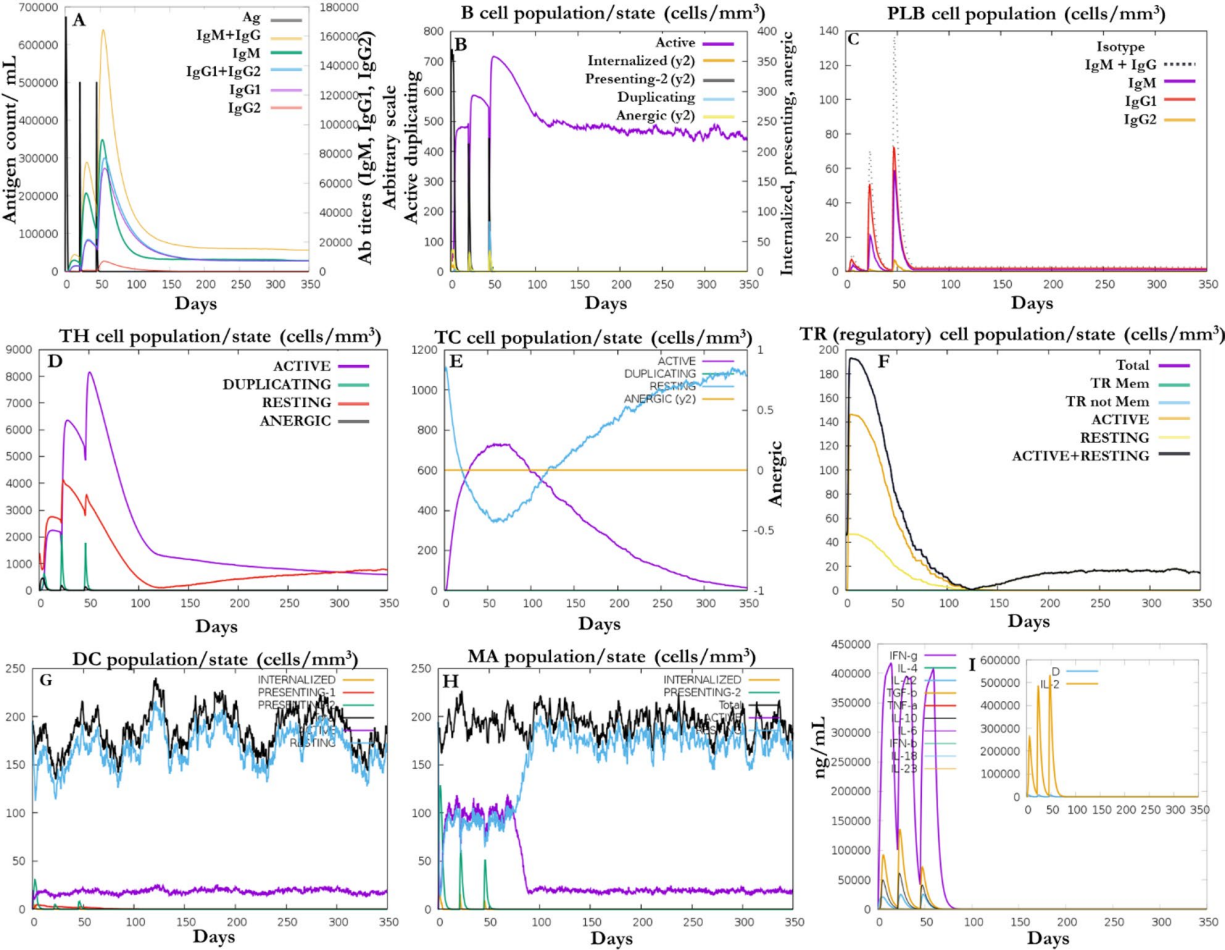
Immunogenic potential of the designed vaccine (DV-1). In response to the exposure of DV-1 after three injections, (**A**) production of immunoglobulins (**B**) active B-cell populations/state; (**C**) plasma B-lymphocytes and their isotypes per state; (**D**) helper T-cell population/state; (**E**) cytotoxic T-cell population/state; (**F**) reduction in the level of T regulatory cells; (**G**) dendritic cell population per state; (**H**) activity of macrophage population/state; (**I**) cytokine level and interleukins (smaller plot) in different states with the Simpson index (dotted line). All units are in cells/mm^3^ in three subsequent immune responses.

The result of the immune simulation after injection of DV-1 is presented in Fig. 10. In response to several exposures of DV-1 to the host immune system, a significantly high level of the secondary and tertiary antibodies was observed (higher antibody detection) when compared to the primary antibodies. Consequently, rapid clearance of the antigen was detected (decrease in antigen concentration) (Fig. 10A). The different immune cell population/state count is presented in Fig. 10B–H. These immune cells were significantly increased with significant memory cell development; however, T-cells were gradually decreased after their initial increase. Similarly, an increase in the levels of cytokines were observed for IFN-ϒ (> 400,000 ng/ml) (Fig. 10). These observations suggest that immune memory development and the vaccine constructs may therefore confer immunity against the dengue virus.

### Conformational stability analysis of the designed vaccines

**Figure 11:**
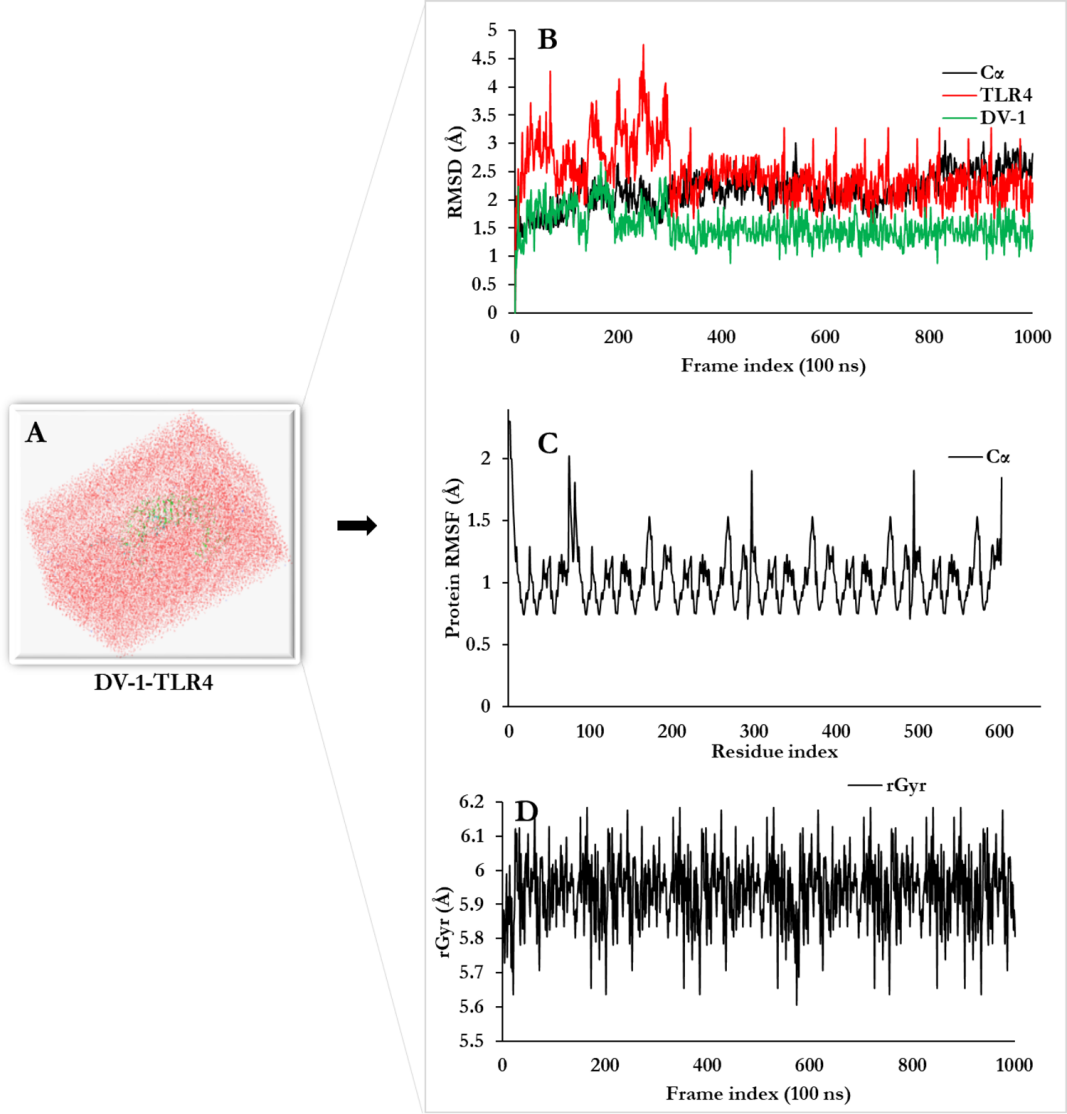
Molecular dynamics simulation of (DV-1-TLR-4 complex at 100 ns (ns). (**A**) Molecular dynamics trajectory (system set-up for the simulation), (**B**) Root Mean Square Deviation of docked complex shows very minimal deviation which reflects the stable microscopic interaction between DV1 and TLR4 molecule (**C**) RMSF-Root Mean Square Fluctuation plot of docked protein complex side chain fluctuation in plot generates peak which reflects the flexibility of side chain of docked protein complex. (**D**) Time evolution of the radius of gyration (rRyr) during 100 ns of MD simulation.

Stability of the complex interactions as well as the dynamic behaviour observed between the designed vaccines and the receptors were elucidated through 100 ns MDs at 300 °K. The statistical parameters such as the root mean square deviation (RMSD), root mean square fluctuation (RMSF), and radius of gyration (rGyr) were used to probe the system for stability and structural adjustment of the vaccine in the binding domains of the selected receptor (Fig. 11A–D). As a measure of the distance between the Cα atoms of superimposed proteins, RMSD was used to evaluate the stability of DV-1 in the binding domain of TLR4 (Fig. 11B). Residual flexibility of DV-1-TLR4 was investigated by RMSF (Fig. 11C) in order to determine the extent and effect of contact deviation on the binding of DV-1 to TLR4. The average RMSF detected for the system was 1.2 Å. In addition; rGyr was used to characterize the secondary elements regular packing in the 3D structure of the DV-1-TLR4 complex and to evaluate complex compactness. The rGyr provides effective information on the tendency of protein structures to expand during MDs^57^. At the time of simulation, the rGyr starts with a sudden increase of 4 ns and remained constant with an average value of 5.98 Å (Fig. 11D). The higher the rGyr the less compact the protein, with less folded stability. Interestingly, the rGyr profile of the system is consistent with the RMSD and RMSF profiles for complex fluctuation^58^.

## Discussion

The use of immunoinformatics approaches in the construction of potent vaccines against various microorganisms especially viruses, are becoming increasingly acceptable as the first line of vaccine development. In recent years, immunoinformatics-guided approaches have been employed in the design of epitope-based subunits for SARS-CoV-2^59–64^. Kar and Srivastava^65^ also identified potential vaccine candidates against *Theileria* parasites and the T-cell epitope vaccine candidate for parasitic helminth infection^66^. Dengue is a mosquito-borne viral infection caused by different serotypes of DENV. These serotypes (DENV-1–4) cross-react immunologically with each other^67^. On a global scale, about three billion people are susceptible to DENV infection, of which approximately 96 million cases occur annually^68^. Individuals with primary infection are highly prone to develop dengue hemorrhagic fever and dengue shock syndrome during secondary infection due to antibody-dependent enhancement^69,70^. To date, there is no specific treatment for dengue fever and the main preventive measure is by vaccination in order to reduce the burden of the disease. Hence, this study aimed at designing novel multi-epitope based DENV vaccine candidates capable of evoking immune responses in DENV infected individuals using computational approaches. Epitope-based vaccines have potential advantages over conventional vaccines and they have received considerable attention in recent years.

DENV protein components were retrieved and used for the multi-epitope based design of dengue vaccine in this study. The selected components were subjected to physical and chemical properties prediction prior to antigenic determination of the sequences. Using the threshold of 0.4, the viral components were classified into antigens and non-antigens using the tumor model of VaxiJen (VaxiJen v2.0). Scores below this threshold were classified as non-antigen and discarded. For accurate component selection, the localization and transmembrane helices of the protein sequences were further determined. This process ascertains the suitability of candidate vaccine selection for experimental validation in vaccine development process^71,72^. The final result of all the structural proteins and NS1, NS3, and NS5 from the non-structural proteins were considered for epitope-based vaccine design in this study. Furthermore, the B and T-cell epitopes were predicted from these antigenic viral components. A good vaccine should be able to induce immunity through the antigen with a durable adaptive immunity. A total of 21 antigenic and non-allergenic epitopes were predicted for both MHC classes, while nine antigenic, non-toxic and non-allergenic epitopes were predicted for the B-cells. The CTL epitopes are responsible for developing durable immunity capable of eliminating circulating virus and infected cells^73^. On the other hand, HTL epitopes are associated with the production of both humoral and cellular immune responses. These epitopes elicit a CD4^+^ helper response for the generation of protective CD8^+^ T-cell memory and activation of B-cells^74^.

The homology modeling of all the predicted multi-epitope vaccines were predicted by Schrödinger and structurally confirmed by three web-based protein structural prediction tools (Phyre^2^, RaptorX, and SCRATCH predictions). The predicted 3D structures of the multi-epitope vaccines were validated using PROCHECK at PDBSum Generate server and ProSA-web. The result of the predicted structural analysis by PROCHECK server is a Ramachandran plot. This plot gives information about the torsional angles (phi (φ)and psi (ψ)) of the residues contained in the input PDB file. The statistics confirmed that approximately 90% of the predicted vaccine residues are in both the most favoured and additional allowed regions while only few residues were detected in the generously allowed and disallowed regions. The model structures were further queried for potential errors by ProSA-Web. This software tool provides a graphical output of the overall and local model quality based on the input and Z-score of the predicted model. The modeled protein structures of Yadav et al.^75^ were reported to be − 5.98 kcal/mol and 6.66 kcal/mol for template and target, respectively. In addition, an investigation of immunogenic properties of Hemolin using an immunoinformatic approach, showed that the Z-score of the modeled protein was − 7.08 kcal/mol^76^. With Z-scores of − 5.26 kcal/mol., − 9.5 kcal/mol and − 2.11 kcal/mol, Droppa-Almeida et al.^77^, Rekik et al.^78^, and Hashemzadeh et al.^79^ concluded that the predicted 3D structures were reliable and of good quality. The overall model quality suggests the qualities of X-ray crystallography with Z-score of − 4.78, − 5.48, − 3.29, and − 4.24 kcal/mol. for the predicted DV-1-DV-4 vaccines, respectively. Overall the results of the validation tools provide evidence that the predicted vaccine structures are acceptable and of good quality. Therefore, the 3D-structures of the predicted vaccines are reliable and were considered for downstream analysis.

Molecular docking is a crucial tool employed in the field of Bioinformatics to explore specific interactions and binding affinities of ligands towards selected receptors based on the lock and key mechanism. In the field of immunoinformatics, protein–protein docking is usually employed to investigate the best, stable and effective vaccine through their poses, interacting atoms and their binding energies. This procedure was used to examine the best possible vaccine construct(s) against the MHC alleles (HLA-A*11–01 and HLA-DRB1*04–01) as well as the immune molecules (TLR2 and TLR4). Several immunoinformatics studies have shown the importance of these receptors in the production of an effective immune response^80–83^. TLRs have a significant function in innate immunity such as immune activation and adaptive immune response. Also, the involvement of TLR2 and TLR4 were previously investigated in the recognition of viral structural proteins leading to inflammatory cytokine production^84^. The docking calculation between the designed constructs (DV-1–4) and the receptors (MHCs and TLRs) showed that the designed vaccines perfectly fit into the binding pockets of the receptors, and therefore, the vaccines may generate stable immune responses. Due to the global energies of the DV-1 and its excellent performance, it was considered for MDs study.

In silico cloning was carried out in a pET28a (+) vector after codon optimization by the JCAT web server in order to avoid codon bias^85^. The CAIs and the GC content of the optimized nucleotides were within the accepted ranges of 0.8–1.0 and 30–70% respectively. These data confirmed that the designed constructs were reliable with efficient expression in the *E coli* strain K12.

C-ImmSim is a data-driven immune prediction algorithm and an agent-based simulator of the immune response previously used to study the immunogenic potentials of predicted vaccines^86^. This algorithm simulates the major functional mammal system components (bone marrow, thymus and lymph node). Since a potent vaccine must imitate the natural immunity induced by antigen with the production of long-lasting adaptive immunity, the response of the immune cells (HTL, CTL, B-cells, dendritic cells, immunoglobulins and cytokines) were reported against the designed vaccines. Immune simulation with DV-1 was predicted to trigger IgG, IgM, B-cell, T-cell and cytokines. Similar to the results of previous immunoinformatics vaccine designed studies^87–89^, the designed vaccines may offer protection against DENV.

The stability of the complex (DV-1-TLR4) was validated by performing a 100 ns at 300° Kelvin MDs using the Schrödinger software package. OPLS_2005 force field and a predefined solvent model of TIP-3P was chosen for the calculation. Furthermore, NaCl was added as (Na^+^ and Cl^−^) and the final salt concentration was 0.15 M in order to simulate the background salt at psychological conditions.From the result obtained, a slight fluctuation of TLR4-vaccine complex was observed from the start of the simulation period to about 300 ns after which there was no major fluctuation in the RMSD profile of the complex. The RMSF profile showed the presence of a highly fluctuating N-terminal region at the beginning of the simulation. This result was consistent with previous studies where immunoinformatics of vaccine–receptor complex stabilization was achieved within a similar time-scale using MDs^59,73,90,91^. The rGyr measures the 'extendedness' of a ligand, and is equivalent to its principal moment of inertia. The rGyr of the DV-1-TLR4 complexes ranged between 5.5 Å to 6.1 Å. This change in rGyr reveals the compactness of the vaccine construct-TLR4 complexes. This result implies that the designed vaccines can strongly interact with immune receptors.

### Limitations

This study highlighted an alternative vaccine approach based on multi-epitope construction of the protein components of dengue genome to handle antigenic complexity. Although the predicted vaccines were recommended based on immunoinformatics strategies and believed to be immunogenic, the extent of protection from dengue infection is unknown. Immunoinformatics approaches are extremely useful to perform in silico studies and can guide laboratory experiments contributing to save time and money. Nonetheless, the next phase is to carry out in vitro immunological assays to validate the predicted vaccines, determine their immunogenicity, and further devise challenge-protection preclinical studies to eventually certify these approaches.

There are some limitations to this current study. First, challenges such as standard benchmark, insufficient prediction methods, and unavailability of precise datasets for different computational analysis are associated with immunoinformatics approach. Secondly, little is known about the pathogenic entry and specific immune-bypass mechanism of dengue within the human host. With this knowledge, the vaccine could be developed more effectively and efficiently to control and/or prevent the pathogenesis of dengue. Thirdly, the proposed vaccine constructs await experimental investigated through *in-vitro* and *in-vivo* bioassays to demonstrate its safety, efficacy, and immunogenicity against dengue by producing an active immunological memory.

On a whole, the application of these results is pending validation in the wet lab experimental models.

## Methodology

**Figure 12:**
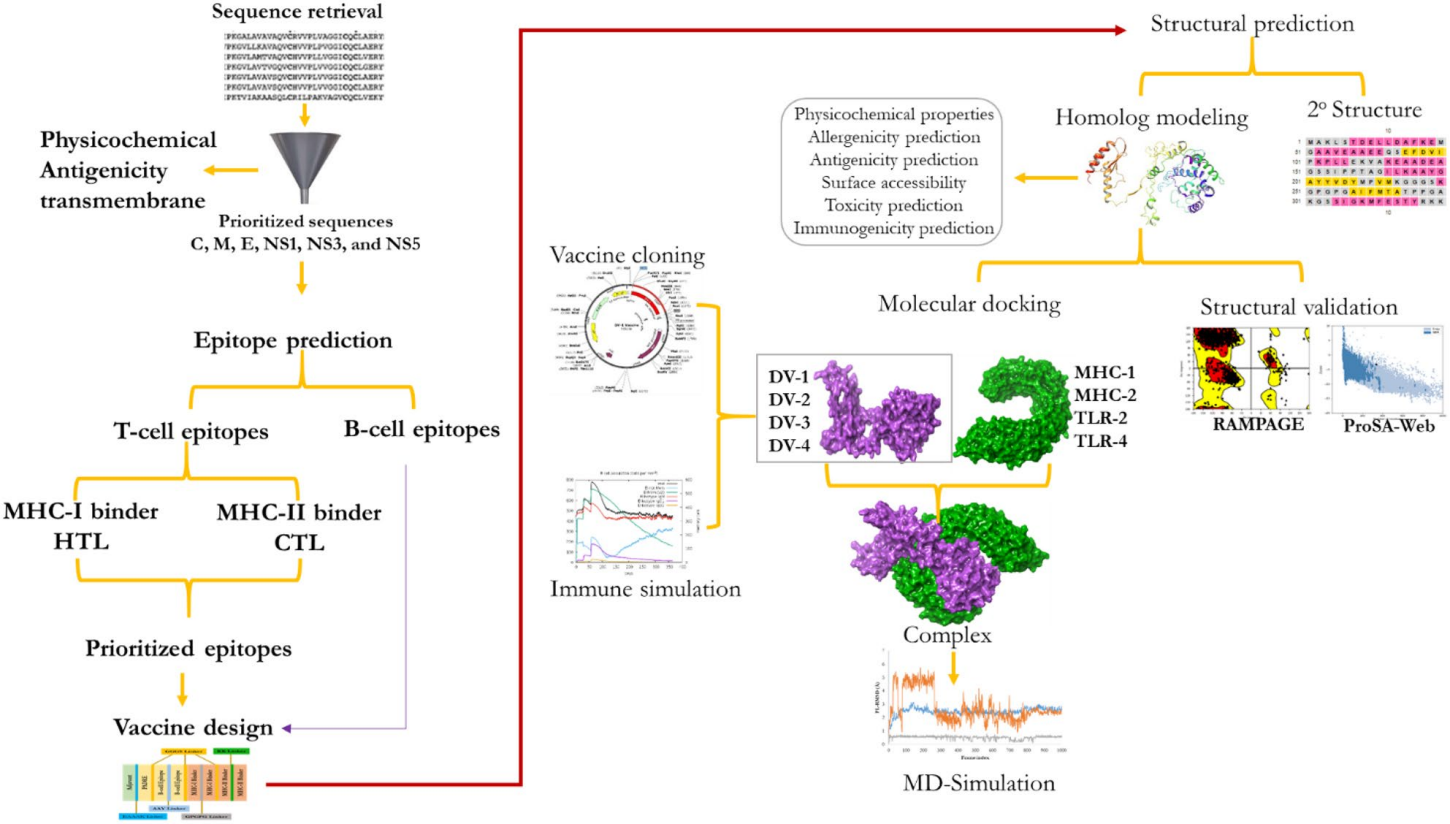
Overview of the study design.

Figure 12 illustrates the stepwise flow of the methodology followed to design a vaccine against DENV.

### Sequence retrieval

All the protein components of DENV 1–4 were retrieved from the virus pathogen resource (VIPR) database at https://www.viprbrc.org/brc/home.spg?decorator=vipr and confirmed with NCBI. These protein components include: the structural proteins (Capsid protein (C), membrane glycoprotein precursor (M), and envelop protein (E)) and the 7 non-structural proteins (NS1, NS2A, NS2B, NS3, NS4A, NS4B and NS5). A single file containing all the sequences was generated in FASTA format.

### Protein selection for vaccine preparation

The complete amino acid sequences of the proteins were retrieved from the NCBI database in the FASTA format. The proteins were subjected to signal peptide analyses using SignalP 4.1 server (http://www.cbs.dtu.dk/services/SignalP/) to discriminate classical secretory and non-secretory proteins. SignalP integrates a prediction of cleavage sites and signal/non-signal peptide predictions based on a combination of several artificial neural networks^92^ signal was reported to outperform other predictors in benchmark tests^93^. Localization predictions were conducted using DeepLoc (http://www.cbs.dtu.dk/services/ DeepLoc/), a template-free algorithm that uses deep neural networks to predict protein subcellular localization using only sequence information with good accuracy^94^.

### Multiple sequence alignment and antigen selection

The NCBI BLAST program at https://blast.ncbi.nlm.nih.gov/Blast.cgi was used to identify highly conserved sequences of the DV. In addition, multiple sequence alignment was achieved using the Multalin server at https://www.multalin.toulouse.inra.fr/multalin. The antigenicity of the viral components (structural and non-structural) were evaluated using VaxiJen 2.0 server (http://www.ddgpharmfac.net/vaxijen/VaxiJen/VaxiJen.html^95^. Finally, the most highly conserved sequences and antigenic peptides were selected for downstream analysis.

### Allergenicity prediction

Allergenicity prediction is a critical step in therapeutics and bio-pharmaceuticals due to their involvement in foods and/or food products. Sequence identity of known allergens is important for predicting the cross-reactive potential of novel proteins and their resistance to pepsin digestion and glycosylation status is used to evaluate denovo allergenicity potential^96^. AllerTOP v2.0 is a bioinformatics tool and web-based server used for protein allergenicity prediction in this study. As outlined by Dimitrov et al.^97^, this tool (https://www.ddg-pharmfac.net/AllerTOP/index.html) uses an auto cross covariance transformation of protein sequences into uniform equal-length vectors. The method was previously applied to quantitative structure–activity relationship studies of peptides with different lengths using five descriptors such as hydrophobicity, molecular size, helix-forming propensity, relative abundance of amino acids and β-strand forming propensity^98^.

### Antigenicity

VaxiJen is a web-based server for prediction of protective antigens, tumour antigens and subunit vaccines^95^. This server accessed at http://www.ddg-pharmfac.net/vaxijen/ was used to determine the best antigenic protein components and to identify epitopes from DENV-4. The viral components’ sequences, predicted epitopes, and constructed vaccines were used as input with ‘tumor’ as selected organism target at a threshold of 4.0.

### B-cell epitope prediction and selection

The B-cell epitopes were predicted using the BCPreds server at http://ailab.ist. psu.edu/bcpred/. This server uses a novel kernel method for predicting linear B-cell epitopes. The epitope length was kept at 20 amino acids with 75% sensitivity. The use of multiple tools in epitope prediction has been reported to improve the rate of true positives in epitopes prediction^99^. Therefore, other prediction servers such as IEDB analysis Resource (http://tools.iedb.org/population)^100^ and the BepiPred-2.0 web server (http://www.cbs.dtu.dk/services/BepiPred/) were used for validation. The method employed by BepiPred-2.0 is more superior to other available tools for sequence-based epitope prediction based on the epitope data derived from solved 3D structures and a large collection of linear epitopes downloaded from the IEDB database. In addition, the proteins were subjected to linear epitope prediction using ABCpred at https://webs.iiitd.edu.in/raghava/abcpred/index.html to compute ABCpred epitope scores at a threshold of 0.5. At this threshold, the tool shows 65.93% accuracy with equal sensitivity and specificity using a window length of 16. Finally, the server, SVMTrip at http://sysbio.unl.edu/SVMTriP/prediction.php was used to improve the prediction performance for the predicted B-cell epitopes**.**

### T-Cell epitope prediction analysis

Following the combined methods of Shey et al.^101^, Ali et al.^53^ and Ullah et al.^48^, the IEDB epitope analysis resource at https://www.iedb.org/home_v3.php was used to predict the MHC class I and MHC class II binding epitopes. Briefly, the MCH class I restricted CD8^+^ cytotoxic T lymphocyte (CTL) epitopes from the antigenic viral components of DENV-4 were predicted using the NetMHCpanEL 4.0 prediction method for HLAA*11–01 allele. The result of this method was further subjected to the SMM method in order to prioritize the selected CTL epitopes using the IC_50_ threshold of 500 nM and percentile ranks of ≤ 0.2. In addition, the 15-mer MCH class II-restricted CD4^+^ helper T lymphocyte (HTL) epitopes were predicted using the NetMHCIIpanEL 4.0 prediction method for HLA DRB1*04–01 alleles. The predicted classes of the MHCs were further screened for antigenicity, non-allergenicity, non-toxicity, highly conservancy and transmembrane topology.

### Toxicity and transmembrane topology analysis

ToxinPred is an in silico software tool used for the prediction as well as the design of both toxic and non-toxic peptides. This tool predicts the toxicity of the epitopes according to their physicochemical properties of the input sequences. With the default settings, the predicted sequences were used as input in order to determine their toxicity. Epitopes that were not predicted as allergens by AllerTOP were subjected to toxicity analysis by the ToxinPred webserver at http://crdd.osdd.net/raghava/toxinpred/. All the peptides predicted as toxic were discarded. In addition, the transmembrane topology experiment of the viral component and the predicted epitopes were evaluated using the TMHMM v2.0 server (http://www.cbs.dtu.dk/services/TMHMM/). This tool is user-friendly for determining the transmembrane topology of epitopes using their sequences as input^102^.

### Analysis of the physicochemical properties and construct solubility

The physicochemical properties of the viral components and the constructed vaccines were predicted by ExPASy ProtParam at https://web.expasy.org/protparam/^103^. Characteristics such as number of amino acids, molecular weight, theoretical pI, half-lives, instability indexes, extinction coefficient, aliphatic index and grand average of hydropathicity were evaluated^104^. Protein-Sol (https://protein-sol.manchester.ac.uk/) was used to evaluate the solubility of the multi-epitope based vaccines^105^.

### Epitope conservancy analysis

The predicted epitopes were subjected to conservancy analysis in order to predict the degree of similarity within the serotypes of dengue virus at sequence identity threshold of ≥ 80%. Using the IEDB analysis resource at http://tools.iedb.org/conservancy/, each of the epitopes predicted were used as input against all the serotypes of dengue (DENV 1–4).

### Vaccine construction

Vaccine construction requires three requisite elements in order to create an efficient molecule capable of eliciting an immune response. These elements include the identified epitopes (the B- and the T-cell epitopes), adjuvants and the spacer sequences. The predicted epitopes from the DENV-4 were selected for the final vaccine construction after the in-house screening processes. In order to amplify the immune system, molecules with immunomodulatory properties were added to the vaccine constructs as safe vaccine adjuvants. The adjuvants were connected to the epitopes using the rigid linker EAAAK. The glycine-proline rich GPGPG linkers were used to space the B-cell and HTL epitopes. In addition, the CTL epitopes were linked using an effective and flexible linker AAY. To prevent the polymorphism of HLA-DR molecules, the Pan DR T Helper Epitope (PADRE epitope) was introduced to the final vaccine sequences. The sequence order of the final vaccines are as follows: Adjuvant-Rigid linker (EAAAK)-PADRE-GGGS-BCL-flexible linker (KK)-BCL-flexible linker (GPGPG)-HTL-flexible linker (GGGS)-HTL-flexible linker (AAY)-CTL. Four vaccines were constructed with three adjuvants namely heparin-binding hemagglutinin (HBHA), beta defensin, and ribosomal protein L7/L12 for the vaccines DV-1 to 4 respectively.

### Homology modelling of the predicted epitopes

The 3D structures of the epitopes and the designed vaccines were modeled using the structure prediction wizard of prime module in the Schrodinger suites v2020-3 using the Maestro v12.3 software interface. Since the process of constructing a 3D atomic-resolution model is based on the principle that evolutionary related proteins share similar sequence and protein structures, homology modeling therefore, relies on high quality sequence alignment and template structure (knowledge-based method of modeling). The predicted epitopes and designed sequences were used as input individually in FASTA format followed by the BLAST homology search. Homology modeling steps such as template recognition and initial alignment, alignment correction, backbone generation, loop and side chain modeling, model optimization and validation were all performed on the 3D predicted models. In addition, the online protein structure predictions; Protein Homology/analogY Recognition Engine V 2.0 (Phyre^2^) at http://www.sbg.bio.ic.ac.uk/phyre2/html/page.cgi?id=index, RaptorX at http://raptorx.uchicago.edu/ContactMap/, and SCRATCH protein predictor at http://scratch.proteomics.ics.uci.edu/ were used to remodel and ascertain the modeled 3D structures of the designed vaccines.

### Secondary structure prediction

PSIPRED V3.3 at http://bioinf.cs.ucl.ac.uk/psipredtest was utilized to predict the secondary structures of the designed vaccines using their primary sequences as input. PSIPRED is a simple and accurate 2D structure prediction method, incorporating two feed-forward neural networks that performs an analysis on output obtained from PSI-BLAST (Position Specific Iterated-BLAST)^106^.

### Structural validation of the modeled vaccines

Following the modified method of Saha and Prasad^107^, the PDBSum module in the EMBL-EBI database (https://www.ebi.ac.uk/) and ProSA-Web at https://prosa.services.came.sbg.ac.at/prosa.php were used to analyze the designed vaccine constructs before and after minimization and refinement in order to validate their purities^108^. Specifically, the PDBSum Generate at http://www.ebi.ac.uk/thornton-srv/databases/pdbsum/Generate.html use the PDB files of the prepared constructs after homology modeling and preparation as input to evaluate purity using the PROCHECK and produces a graphical output in the form of a Ramachandran plot^109^. The higher the percentage of atoms in the most favored region the greater the resolution and purity and the models can be used for molecular docking calculations.

### Receptor selection

It is crucial for designed vaccines to interact with immune cells in order to confer the stability of the immune response. The molecular docking calculation study was performed to investigate these interactions using the Major histocompatibility complex (MHCs) receptors as well as the Toll-like receptors (TLRs). Following previously methods of receptor selections^64,101,110^, the crystal structures of MHC class I allele (HLA-A*11–01) and MHC class II allele (HLA DRB1*04–01) with PDB IDs, 5WJL and 5JLZ were retrieved from the Protein Data Bank (PDB) at https://www.rcsb.org/. In addition, the 3D structure of TLR4 dimer and TLR2 (PDB ID: 3FXI and PDB ID: 2Z7X) were retrieved for additional docking purposes. These proteins were refined, minimized and optimized using the protein preparation wizard in the Schrodinger suite and were further considered for molecular docking analysis with the multi-epitope constructed vaccines^111^.

### In silico docking calculation

To inspect the binding affinity of the modeled vaccines to MHCs and TLRs molecules, the designed vaccines were docked against the selected receptors using the Prime protein–protein docking module in Schrodinger^112^. The binding score and the atomic contact energy were analyzed using the PATCHDOCK server at https://bioinfo3d.cs.tau.ac.il/PatchDock/php.php while the refinement of the generated poses including the global energy were carried out using the FIREDOCK server at http://bioinfo3d.cs.tau.ac.il/FireDock/php.php.

### Optimization of designed vaccine candidate

Reverse translation and codon optimization were performed using the Java Codon Adaptation Tool (JCat) server (http://www.jcat.de/) in order to express the multi-epitope vaccine construct in a selected expression vector^113^. Codon optimization was performed in order to express the final vaccine construct in the *E. coli* (strain K12) host, as codon usage of *E. coli* differs from that of ‘homo sapiens’, where the sequence of the final vaccine construct is derived. In the additional options section, the ‘avoid the rho-independent transcription termination’, ‘avoid prokaryote ribosome binding site’, and ‘avoid restriction enzymes cleavage sites’ were all checked. The JCat output includes the codon adaptation index (CAI) and GC content (%), which can be used to assess protein expression levels. CAI provides information on codon usage biases; the ideal CAI score is 1.0 but > 0.8 is considered a good score^114^. The GC content of a sequence should range between 30–70%. GC content values outside this range suggest unfavorable effects on translational and transcriptional efficiencies^53^. To clone the optimized gene sequence of the final vaccine constructs in *E. coli* pET-28a (+) vector, *Xho*I and *Nde*I restriction sites were added to the N and C-terminals of the DNA sequences of the predicted vaccines, respectively. Finally, the optimized DNA sequences (with restriction sites) were inserted into the pET-28a (+) vector using SnapGene tool to ensure vaccine expression.

### Immune simulation of the designed vaccines

In order to understand the immune system relative to the predicted vaccines, C-ImmSim at http://150.146.2.1/C-IMMSIM/index.php?page=1 were employed to assess the molecular binding of immune complexes. The principle of operation of this server is based on the position-specific scoring matrix and machine learning approaches to predict subunit vaccine and binding interactions. Following the clinical vaccine recommended procedures^115,116^, and immune simulation methods previously reported^89,101,117,118^, three injections were administered for a total of 1000 steps of simulation using the designed vaccine sequences as input.

### Molecular Dynamic simulation (MDs)

MDs of the designed vaccines of the immunogenicity were characterized by the system build and molecular dynamic Desmond package in Schrödinger. Prior to simulation, the docked complexes were prepared using the system builder module in Maestro v12.4. The optimized potentials for the liquid simulations OPLS-2005 force field were used to determine the receptor interactions which were solvated with the simple point charged (TIP-3P) water model^119,120^. The orthorhombic water box was used to create a 10 Å buffer region between the atoms on the receptors and box sides. The volume of the box was minimized and the overall charge of the system was neutralized by adding Na^+^. The temperature and pressure were kept constant at 300° Kelvin and 1.01325 bar using the Nose–Hoover thermostat^121^ and Martyna–Tobias–Klein barostat methods. The simulations were performed using NPT ensemble by considering the number of atoms, pressure and timescale and the simulation time at 100 ns. The MD results were analyzed by simulation interactions diagram module and MS-MD trajectory analysis.

## Conclusion

In this study, immunoinformatics approaches were employed in the development of potential vaccine candidates against DENV considering their ease of use in experimental investigations. Since the only viable defense for DENV infection is a vaccine; four potential vaccines were designed and predicted to elicit specific immune responses in individuals with dengue infection. The results of this study are based on computational approaches. Therefore, it is required that both *in-vivo* and *in-vitro* laboratory experiments be carried out to ascertain the results of this study.

## Data Availability

All data generated or analysed with respect to this study are included in the submitted manuscript. The sequences of the protein analysed can be retrieved from NCBI (https://www.ncbi.nlm.nih.gov/), UniProt (https://www.uniprot.org/), and Protein Data Bank (https://www.rcsb.org/) using their accession numbers.
